# Effects of Dietary Vitamin E Supplementation in Bladder Function and Spasticity during Spinal Cord Injury

**DOI:** 10.3390/brainsci8030038

**Published:** 2018-02-26

**Authors:** Kathia Cordero, Gemma G. Coronel, Miguel Serrano-Illán, Jennifer Cruz-Bracero, Johnny D. Figueroa, Marino De León

**Affiliations:** Center for Health Disparities and Molecular Medicine, Department of Basic Sciences, Loma Linda University School of Medicine, Loma Linda, CA 92350, USA; kacordero@llu.edu (K.C.); gcoronel@ucsc.edu (G.G.C.); mserranoillan@llu.edu (M.S.-I.); jcb5288@gmail.com (J.C.-B.); jfigueroa@llu.edu (J.D.F.)

**Keywords:** spinal cord injury, Vitamin E, bladder dysfunction, H-reflex, oligodendrocytes

## Abstract

Traumatic spinal cord injury (SCI) results in debilitating autonomic dysfunctions, paralysis and significant sensorimotor impairments. A key component of SCI is the generation of free radicals that contributes to the high levels of oxidative stress observed. This study investigates whether dietary supplementation with the antioxidant vitamin E (alpha-tocopherol) improves functional recovery after SCI. Female adult Sprague-Dawley rats were fed either with a normal diet or a dietary regiment supplemented with vitamin E (51 IU/g) for eight weeks. The rats were subsequently exposed either to a contusive SCI or sham operation, and evaluated using standard functional behavior analysis. We report that the rats that consumed the vitamin E-enriched diet showed an accelerated bladder recovery and significant improvements in locomotor function relative to controls, as determined by residual volumes and Basso, Beatie, and Bresnaham BBB scores, respectively. Interestingly, the prophylactic dietary intervention did not preserve neurons in the ventral horn of injured rats, but it significantly increased the numbers of oligodendrocytes. Vitamin E supplementation attenuated the depression of the H-reflex (a typical functional consequence of SCI) while increasing the levels of supraspinal serotonin immunoreactivity. Our findings support the potential complementary use of vitamin E to ameliorate sensory and autonomic dysfunctions associated with spinal cord injury, and identified promising new cellular and functional targets of its neuroprotective effects.

## 1. Introduction

Primary mechanical injury to the spinal cord sets in motion a complex cascade of secondary harmful events that result in serious neurological dysfunction and paralysis. Following the initial lesion, events associated with a secondary injury can last several days and weeks after injury. Disorders associated with the secondary injury include dramatic metabolic alterations [1,2,3,4,5,6,7,8,9], a general increase in oxidative stress and inflammation [10,11,12,13,14,15,16,17,18,19,20], demyelination [20,21,22,23,24,25], and apoptosis [26,27,28,29]. During the first week after injury there is an extensive loss of neurons and oligodendrocyte [23,28,29,30,31,32,33], lipid peroxidation [10,34], and axon degeneration [14]. The production of free radicals during this period is believed to contribute to these detrimental outcomes by disrupting cell membranes, causing organelle dysfunction, and disturbing calcium homeostasis [34]. The free radical production peaks at 12 h after the initial injury and it remains elevated for at least 1 week after injury. Examples of elevated free radicals after injury are hydrogen peroxide, hydroxyl radical, and peroxynitrite radical [14,35]. Previous studies have shown a reduced antioxidant defense after SCI [36] and antioxidant agents such as tempol have shown to be neuroprotective in the context of SCI [37].

Previous reports from our laboratory showed that dietary prophylaxis with omega-3 lipids is protective during SCI. For instance, dietary omega-3 polyunsaturated fatty acids (O3PUFAs) modulated multiple pathways that contribute to secondary acute and chronic damages following SCI [38,39]. Administration of O3PUFAs restored the spinal cord lipid homeostasis, confers neuroprotection, prevents sensorimotor dysfunction and neuropathic pain, and facilitates locomotor recovery following acute and chronic SCI when administered before and during SCI [38,39,40]. Another nutrient with demonstrated antioxidant capability that could be important in stimulating recovery following SCI injury is vitamin E. Previous studies using dietary vitamin E supplementation for 5 days prior to the SCI in cats found a reduction of arachidonate and prostanoids [41] associated with less ischemia [42] and enhanced locomotion recovery [43]. Further, dietary vitamin E for 8 weeks before SCI decreased reactive oxygen species (ROS) while improved locomotion, blood flow and spinal cord evoked potentials, and decreased ROS in rats [44]. While these studies did not use the standardized scale to measure locomotion after SCI, the Basso, Beattie, and Bresnahan (BBB) scale, their findings provided support for a promising neuroprotective role of vitamin E. Further, dietary vitamin E supplementation for 14 days after injury was shown to improve BBB scores [45,46] potentially involving the inhibition of lipid peroxidation products such as thiobarbituric acid reactive substances and malondialdehyde [44,47,48]. Thus, prior studies suggest a neuroprotective role of vitamin E during SCI, but little is known about potential targets, primary cellular processes involved and whether it can affect other primary functions affected by SCI. For instance, it is unknown whether dietary prophylaxis with vitamin E can specifically address specific impairments associated with SCI such as bladder dysfunction and spasticity. These processes can be especially sensitive to oxidative stress, serotonin levels or adequate number of oligodendrocyte. The present study evaluates the effects of two months dietary exposure to vitamin E supplementation on selected physiological outcomes and examined potential mechanisms. The underlying hypothesis of the current study is that a balanced antioxidant dietary regimen containing adequate levels of vitamin E may enhance the ability of the spinal cord to response to traumatic injury. The data suggest that dietary vitamin E supplementation significantly improved BBB locomotor scores following SCI. Further, we also report for the first time that prophylactic vitamin E administration stimulates bladder recovery, inhibited H-reflex depression, supraspinal serotonin levels and preserved oligodendrocytes survival.

## 2. Materials and Methods

### 2.1. Animals

Experimental procedures were compliant with the Loma Linda University School of Medicine regulations and institutional guidelines consistent with the National Institutes of Health Guide for the Care and Use of Laboratory Rats. All animal work reported in this manuscript was performed under the approval the Loma Linda University Health Institutional Animal Care and Use Committee approval number 8170021. A total of 60 female Sprague-Dawley rats weighing 182–212 g were acquired from Charles River Laboratories (Portage, MI, USA). The female rats were housed in individual cages with food and water ad libitum with alternated exposure of light and dark periods of 12 h.

### 2.2. Study Design

**Figure 1:**
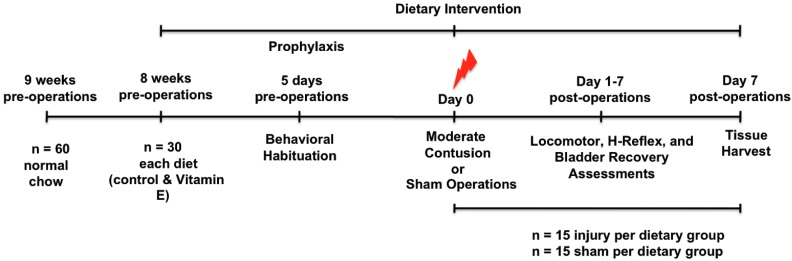
Timeline showing the vitamin E diet supplementation schedule and the time points of behavioral assays, surgical procedures, and tissue sample collection.

The female rats were acclimatized for 1 week after arrival at the animal care facility and were randomly divided into two main groups: (Group 1) rats on the control diet (*n* = 30) and (Group 2) rats on the vitamin E-enriched diet (*n* = 30) (Figure 1). After 8 weeks, the rats were further categorized based on their dietary and surgical interventions: Group 1a was the control diet/sham operated group (*n* = 15); Group 1b was the control diet/spinal cord injured group (*n* = 15); Group 2a was the vitamin E-enriched diet/sham operated group (*n* = 15); and Group 2b was the vitamin E-enriched diet/spinal cord injured group (*n* = 15). Dietary interventions were continued after surgery for 1 week post-operation (wpo). During the first week after operation functional recovery and behavioral analysis was performed in a blinded manner. Spinal cord tissue was collected for analyses at 1 wpo. We opted for not including a group with a vitamin-E enriched diet after injury only because it has been shown the number of pellets eaten the week following spinal cord injury, the acute phase, substantially decreases [39,40]. In a setting where the sub-acute and/or chronic effects of a vitamin-E enriched diet post injury were being studied, such group would definitely contribute to the study, but not in our acute phase, study.

### 2.3. Diets

American Institue of Nutrition (AIN)-93G-based custom isocaloric diet formulations were prepared with modified fat compositions (Bio-Serv, Frenchtown, NJ, USA). The amount of dietary fat was supplied as either soybean oil (control diet) or vitamin E-enriched diet and it was approximately 6% of dry weight. They were both stored in a refrigerated area. Gas chromatography and mass spectrometry analysis showed principal nutrients in the diet as follow: (i) the amount of vitamin E in the control diet was 0.0816 IU/g and the vitamin E-enriched diet had 51 IU/g; (ii) total saturated fat was 1.13 g/100 g and 1.00 g/100 g, total monounsaturated fat was 1.61 g/100 g and 1.49 g/100 g, total polyunsaturated fat was 4.09 g/g and 4.34 g/g for control diet and vitamin E-enriched diet, respectively; (iii) the percentage for kcal carbohydrates was 64.7 and 60.5, the percentage for kcal protein was 18.8 and 21.1, and the percentage for kcal fat was 16.5% and 18.4% for control diet and vitamin E-enriched diet, respectively. During the study rats consumed 15–20 pellets per day. Each pellet weighted approximately 1 g and it contained 51 IU/g of vitamin E. We calculate that each rat consumed 765–1020 IU of vitamin E per day. The amount of vitamin E that has been shown to slow down disease progression in Alzheimer’s disease in human studies is 2000 IU per day (REF [49,50,51]. The relationship between how many vitamin E IUs are required for a protective effect in rats vs. humans is not linear since the average rat weight is 0.3 kg and the average human weight is 70 kg. This can be explained by the higher absolute energy expenditure in rats (600 kJ per kg BW in a 0.2-kg rat) compared to humans (138 kJ per kg BW in a 70-kg human). For more details on the metabolic weight relationship between rats and human, please see our previous publication for a more extensive explanation [39,40]). 

### 2.4. Surgical and Post-Operative Procedures

The female rats were on dietary pretreatment for eight weeks. Following the treatment, the rats were anesthetized with a combination of ketamine/xylazine (80 mg/kg and 10 mg/kg, respectively). The New York University (NYU) Impactor was used to generate the spinal cord lesions [52]. This device causes the necessary trauma to induce bladder dysfunction, which suggests this model is appropriate for evaluating the therapeutic potential of our intervention ([53,54]). The procedure includes exposing the spinal cord by removing the skin and the muscles overlying the spinal column. Laminectomy was performed at the T9–T10 level and the T8 and T12 spinal processes were clamped to the Impactor exposing the dorsal surface of the spinal cord. Next, the dorsal surface was then subjected to weight drop impact with a 10-g rod released from a height of 12.5-mm. Sham rats were not subjected to weight drop impact and only received a laminectomy. The female rats body temperature was maintained at 37 °C during the whole procedure. After laminectomy or weight drop impact, muscle layers were carefully sutured and skin layers closed. Crede’s maneuver was performed three times per day to express the bladders of the injured rats until voiding reflexes were restored. All rats were injected twice per day with Cefazolin (from Bristol Myers Squibb, New York, NY, USA; 25 mg/kg, s.q.) for 5 consecutive days and Buprenex® (also known as buprenorphine; from Reckett and Colman Pharmaceuticals, Inc. Richmond, VA, USA; 0.05 mg/kg, s.q.) for 3 consecutive days. Rats were sacrificed 1 week post-operation and the spinal cord tissue was dissected and collected for immunohistochemical analysis.

### 2.5. Behavioral Evaluation of Spontaneous Locomotion

The Basso-Beattie-Bresnahan (BBB) scale is a 22-point (0–21) measurement scale that evaluate the rat’s spontaneous open-field locomotion [55]. First, the rats were acclimatized in an empty-plastic black pool simulating an open field environment for five daily sessions 1 week before SCI. After SCI, rats were videotaped weekly in the open field locomotion test. In a blind manner, two observers independently assessed: (1) locomotive function, (2) joint movement, (3) paw placement and rotation, (4) paw coordination, (5) tail position, and (6) trunk position and stability. The average score from both blind observers for each hind paw was used for analyses. According to this measurement scale, a score of 0 is given to a completely paralyzed rat, scores between 1 and 8 are given to rats with increasing joint movements without weight support (recovery stage 1), scores between 9 and 13 are given to rats with abnormal locomotion that are able to produce weight supported steps (recovery stage 2), scores between 14 and 20 are given to rats that have reached graded coordination patterns, paw position, and trunk stability (recovery stage 3), and a score of 21 is given to normal (and sham) rats with no dysfunction in locomotion.

### 2.6. H-Reflex Recording

The rats were placed on a heated (approximately 37 °C) metal platform. The Hoffmann’s reflex (H-reflex) was recorded from the plantar muscles of the hind paw, with the active needle electrode (30-gauge) inserted between the fourth and fifth metatarsals, and the reference electrode inserted in the skin of the fifth digit. To elicit the H-reflex and study its rate sensitivity, the tibial nerve at the ankle was stimulated for 0.1 ms at 0.1, 0.3, 1, 2, 3 and 5 Hz using the TECA Sapphire 4ME EMG unit. The cathode needle was inserted subcutaneously at the ankle, just above the heel, and the anode needle was inserted subcutaneously at the plantar surface of the heel. The intensity of the stimulus was adjusted to elicit the maximal consistent H-wave amplitude. The recorded signal was passed to a differential amplifier and bandpass filtered at 0.1 Hz and 10 kHz. The analog signal was then sent to an A/D converter and the digital waveform (recorded at 30 kHz) and stored. Sixteen consecutive waveforms were collected at each frequency. The differences in amplitudes of M- and H-waves as determined by the peak-to-peak values of each waveform were used to calculate the H-reflex depression. The investigator was blinded to the experimental groups during data recording and analyses.

### 2.7. Autonomic Bladder Control Recovery

Crede’s maneuver was used to express the bladders of the injured rats. We used this technique to express the bladder three times a day until bladder function was restored. Each morning we counted how many rats in each diet regained bladder control before 7 dpi and after 7 dpi. Bladder function is restored when the maximum amount of collected volume is 500 μL or less for at least two consecutive days.

### 2.8. Immunohistochemistry Studies and Microscopy

Spinal cord coronal sections were dried at room temperature for 10–15 min, washed with Phosphate Buffer Saline (PBS) for 5 min, and post-fixed with 4% PFA for 10 min. The sections were blocked with 20% Bovine Serum Albumin (BSA) for 2 h at room temperature, and incubated at 4 °C in antibody solutions containing either mouse anti-Neuronal Nuclei (NeuN) monoclonal antibody (clone A60, 1:100; from Millipore, Billerica, MA, USA), mouse anti-adenomatous polyposis coli (APC)-7 monoclonal antibody (clone CC-1, 1:200; from Calbiochem, San Diego, CA, USA), and rabbit polyclonal anti-5HT antibody (1:500; from Abcam, Cambridge, MA, USA) to examine the immunoreactivity (IR) and cell numbers of neurons, mature oligodendrocytes, and IR of serotonin. On the next day, the sections were incubated with Alexa Fluor^®^488 or 594-conjugated donkey anti-mouse (1:500; from Invitrogen, Carlsbad, CA, USA). Control slides were incubated without primary antibodies to further confirm the specificity of the IR. The slides were examined with a BIOREVO BZ-9000 fluorescent microscope (Keyence, Itasca, IL, USA) 

### 2.9. Statistical Analyses

Data are presented as mean +/– SEM. One-way analysis of variance (ANOVA), followed by Bonferroni post-hoc comparisons, was used to determine the effect of spinal cord injury and vitamin E supplementation on open-field locomotion scores, H-reflex depression, NeuN+ cell counts, APC+ cell counts, and 5HT IR. Unpaired *t*-test was used to analyze the difference of APC+ cell counts between uninjured and injured vitamin E groups. Fisher’s Exact Test was used to determine the effect of vitamin E in bladder recovery during the first week post-SCI. Statistical analyses were performed using Prism 6 Software (GraphPad Software Inc., San Diego, CA, USA). Outliers were identified using the Grubbs’ method, also known as ESD (extreme studentized deviate). Only one rat was excluded from the study after using these exclusion methods. Statistical differences were considered significant at *p* <0.05.

## 3. Results

### 3.1. Dietary Vitamin E Improves Locomotor Recovery after SCI

**Figure 2:**
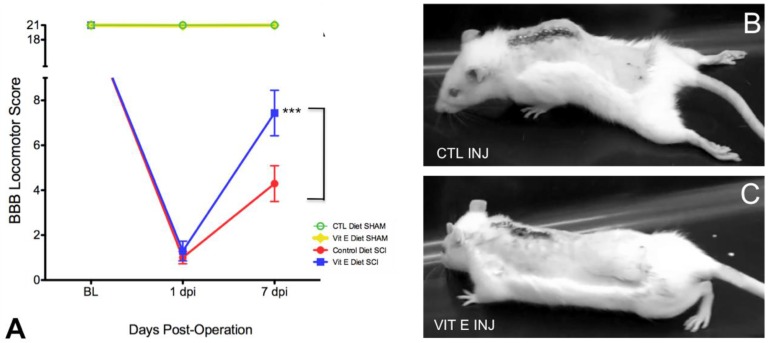
Beneficial effects of dietary vitamin E prophylaxis on the hindlimb neurological function of rats after a moderate injury, as assessed by the Basso-Beattie-Bresnahan (BBB) locomotor rating scale. (**A**) Effects of dietary vitamin E prophylaxis on locomotor function. Time (in days) following pretreatment and spinal cord compression (injury) is shown on the horizontal axis, BBB locomotor rating scores are shown on the vertical axis. Injured rats in the dietary vitamin E prophylaxis group had higher BBB scores of at least 7 when compared to controls. Open-field locomotion still images from control diets- (**B**) and dietary vitamin E prophylaxis rats (**C**) at 7 days post-injury (dpi). Mean dietary vitamin E prophylaxis scores revealed that most rats exhibited extensive movements of three joints with dorsal-stepping patterns at 7 dpi. Bonferroni test analysis was carried out to determine the statistically significant differences between diet treatments. Error bars represent means ± standard error of the mean (CTL INJ versus VIT E INJ *** *p* < 0.001; CTL INJ, *n* = 14; VIT E INJ, *n* = 12). CTL = control, VIT = vitamin, INJ = injury

One week after behavioral habituation period, female Sprague-Dawley rats were provided ad libitum access to one of two diets: control diet or vitamin E-enriched diet (See Table 1 for detailed diet composition). The rats remained on their assigned diets for a total of 8 weeks before injury and 1-week post-SCI. Functional recovery was assessed during the first week after injury. At the end of the study, the spinal cords were harvested and tissues were used for immunohistochemical studies (Figure 1 summarizes the study timeline). The BBB locomotor grading scale was used to evaluate the effects of vitamin E pre-administration on the functional recovery of injured rats. A video recording of each subject’s performance in the open field was obtained at 1 and 7 days post-injury (dpi). We found significant improvements in locomotor behavior in rats from dietary vitamin E prophylaxis group (*n* = 12) compared to rats fed with control diet at 7 dpi (*n* = 14) (Figure 2A) (F (3, 4190) = 367.2, CTL INJ, *n* = 14 versus VIT E INJ, *n* = 12 *** *p* < 0.001). Still images from representative rats fed control diets shows limited movement, dragging of hindlegs, and slight movement of hindlimb joints (Figure 2B) at 7 dpi. Interestingly, rats fed with the vitamin E enriched diet exhibited signs of intermediate locomotor recovery, as indicated by their ability to generate extensive movements of all three joints with dorsal-stepping patterns (Figure 2). 

### 3.2. Dietary Vitamin E Prophylaxis Restores H-Reflex Depression at 7 dpi at 5 Hz.

**Figure 3:**
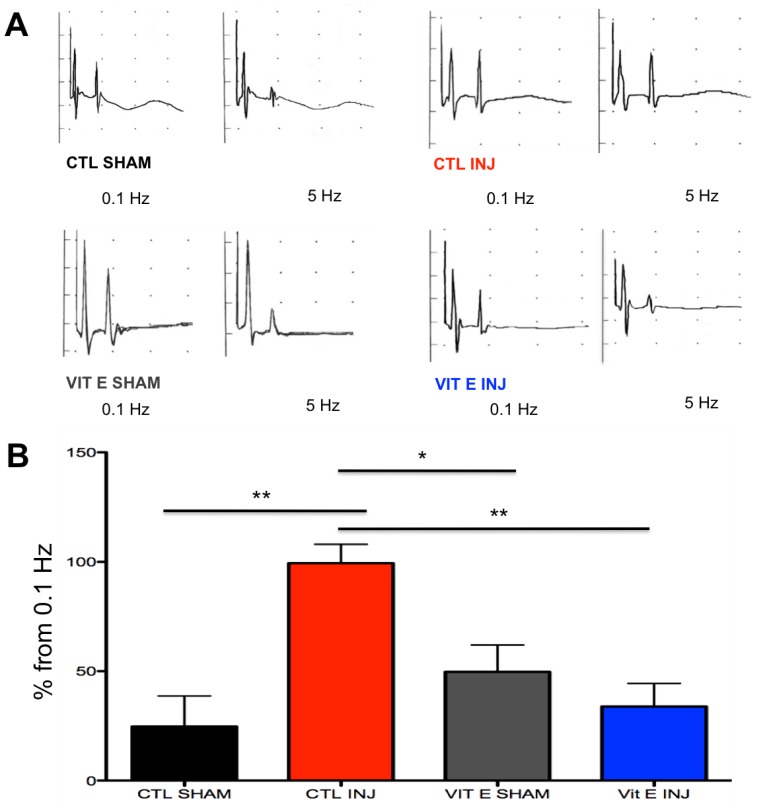
Dietary vitamin E prophylaxis restores H-reflex depression at 7 dpi at 5 Hz. Increased amplitudes on the y axis at 5 Hz indicate less H-reflex rate depression, whereas decreased amplitudes indicate more rate depression. The H-reflex depression after increased frequency (i.e., 5 Hz) was abnormal in rats on a control diet after SCI but it was restored in rats on dietary vitamin E prophylaxis after SCI at 7 dpi (**A**). Higher percent changes from 0.1 Hz at 5 Hz indicate abnormal H-reflex rate depression after SCI in rats on a control diet but not in rats on dietary vitamin E prophylaxis at 7 dpi where there was a lower percent change from .1 Hz (**B**). (** *p* < 0.01 CTL SHAM vs. CTL INJ and CTL INJ vs. VIT E INJ and * *p* < 0.05 VIT E SHAM vs. CTL INJ; CTL SHAM *n* = 6, CTL INJ *n* = 6, VIT E SHAM *n* = 6, VIT E INJ *n* = 7)

Hyperreflexia and spasticity are common complications in SCI with limited availability for safe and effective treatment. A central mechanism in spasticity is hyperexcitability of the spinal stretch reflex. This reflex presents symptomatically as a velocity-dependent increase in tonic stretch reflexes and exaggerated tendon jerks, resulting in functional deficits, pain, and musculoskeletal deformities. Given that the Hoffmann’s reflex (H-reflex) can be evoked in rats, we chose to study the efficacy of our dietary intervention to restore this reflex. As expected, we found reduced H-reflex depression as the stimulation frequency was increased in SCI rats. Notably, the rats that consumed the vitamin E-supplemented diet showed improved H-reflex depression, suggesting less SCI-induced hyperreflexia. Sham-operated rats exhibited normal H-reflex depression in both dietary groups (control diet and vitamin E prophylaxis) (Figure 3A). 

Vertical bars in Figure 3B represent the size of the difference between the M-wave and the H-wave at 5 Hz compared to low frequency stimulation (0.1 Hz). A lower vertical bar on the *y*-axis indicates a bigger difference between the M-wave and H-wave (the latter being smaller), thus showing more H-reflex depression. A higher vertical bar on the *y*-axis value indicates a smaller difference between the M-wave and the H-wave at 5 Hz compared to 0.1 Hz thus less H-reflex depression. 

In uninjured control rats and uninjured rats that received and vitamin E diet, the H-wave steadily decreased with increasing stimulus frequency with the maximum frequency being 5 Hz. However, in injured rats that received a control diet before SCI, the H-wave depression was inhibited showing how the H-reflex was less sensitive to increased stimulus frequency at 5 Hz. As shown in Figure 3, injured rats that received a vitamin E diet before SCI showed a decrease in H-wave depression similarly to uninjured control rats and uninjured vitamin E rats. This finding is indicated by a lower H-wave amplitude at 5 Hz compared to injured rats from a control diet (Figure 3) (F (3, 20,287) = 8.231, (** *p* < 0.01 CTL SHAM vs. CTL INJ and ** *p* < 0.01 CTL INJ vs. VIT E INJ and * *p* < 0.05 VIT E SHAM vs. CTL INJ; CTL SHAM *n* = 6, CTL INJ *n* = 6, VIT E SHAM *n* = 6, VIT E INJ *n* = 7). In summary, H-reflex depression became abnormal after SCI only in injured rats that received a control diet before SCI but not in injured rats that received a vitamin E diet before SCI.

### 3.3. Beneficial Effects of Dietary Vitamin E Prophylaxis on Autonomic Function after Contusion Injury

**Figure 4:**
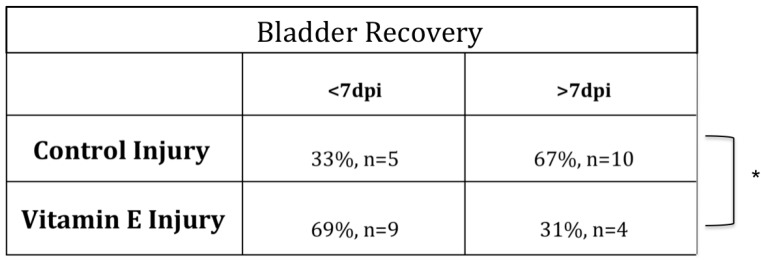
Beneficial effects of dietary vitamin E prophylaxis on autonomic function after SCI. Residual urine volumes (mL) differed significantly between control and dietary vitamin E prophylaxis pre-treated groups at 7 dpi. For each rat, the number of days needed to attain full autonomic recovery was defined as residual volume of 0.5 mL for 2 or more consecutive days. Dietary vitamin E prophylaxis resulted in fewer days (<7) to attain full bladder recovery (* *p* < 0.05 CTL INJ vs. VIT E INJ)

SCI results in a period of distinctive bladder dysfunction [56,57,58,59,60,61,62,63]. Manual collection and quantification of the residual urine volume was done to assess whether dietary vitamin E prophylaxis show efficacy in accelerating autonomic bladder recovery (Figure 4). For each rat, the number of days needed to attain full autonomic recovery was defined as residual volume of 0.5 mL or less for 2 or more consecutive days, as previously reported by our group. We found that the rats that consumed the vitamin E-enriched diet restored bladder function faster relative to controls. (Fisher’s Test analysis of contingency tables * *p* < 0.05 CTL INJ *n* = 15, VIT E INJ *n* = 13). 

### 3.4. Dietary Vitamin E Does Not Preserve Neurons at 1 Week after Spinal Cord Injury (SCI)

**Figure 5:**
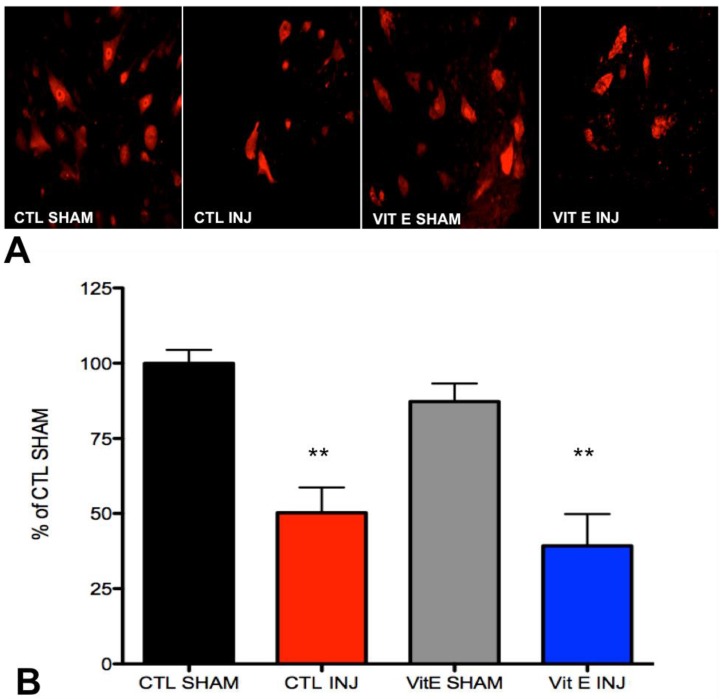
Vitamin E does not preserve neurons at 1 week after SCI. (**A**) Expression of neuronal markers was quantified in the ventral gray matter (VGM). (**B**) Manual quantification of cell numbers and normalization to controls shams revealed decreased numbers of NeuN+ cells in the VGM in injured control rats and injured vitamin E fed rats. The number of NeuN+ cells in the ventral gray matter of the spinal cord from injured control rats was not significantly different from injured vitamin E fed rats. Bonferroni test analysis was carried out to determine the statistically significant differences between diet treatments. Error bars represent mean ± standard error of the mean (** *p* < 0.01, CTL SHAM, *n* = 6 vs. CTL INJ, *n* = 6; CTL SHAM, *n* = 6 vs. VIT E INJ, *n* = 7; *p* > 0.05, CTL INJ, *n* = 6 vs. VIT E INJ, *n* = 7).

To determine the number of motor neurons in the ventral gray matter of the spinal cord, immunohistochemical analyses were performed during the first week following SCI. This represents a critical period in apoptotic cell death in SCI models [26,27,28,29,30]. Consistent with previous findings, we found a significant decrease in the number of neuronal nuclei positive (NeuN+) cells in the ventral gray matter of injured rats when compared to uninjured sham rats at 1-week post-SCI. Interestingly, the dietary intervention did not result in increased NeuN+ cells after SCI. Representative images from sections labeled anti-NeuN (Figure 5A) did not show significant differences in motor neuron cell counts when comparing vitamin E-fed rats with controls at 7 dpi (*p* > 0.05) (F (3, 13,256) = 13.19, ** *p* < 0.01, CTL SHAM, *n* = 6 vs. CTL INJ, *n* = 6; *** *p* < 0.001 CTL SHAM, *n* = 6 vs. VIT E INJ, *n* = 7; *p* > 0.05, CTL INJ, *n* = 6 vs. VIT E INJ, *n* = 7).

### 3.5. Dietary Vitamin E Preserves Oligodendrocytes Following SCI

**Figure 6:**
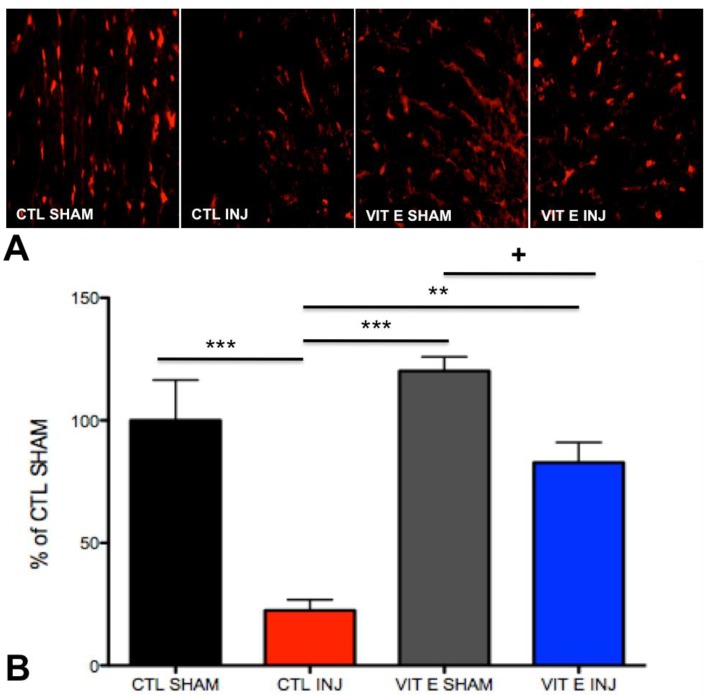
Vitamin E preserves oligodendrocytes at 1 week after spinal cord injury. (**A**)Expression of oligodendrocyte markers was quantified in the white matter of the spinal cord. (**B**) Manual quantification of cell numbers and normalization to control shams revealed decreased numbers of APC+ in the white matter of injured control rats, but not in injured vitamin E fed rats. There was a statistically significant increase of APC+ cells in vitamin E fed rats compared to injured control rats. Bonferroni test analysis was carried out to determine the statistically significant differences between diets. There was a significant difference in APC positive cells between uninjured rats in the vitamin E-enriched diet and injured vitamin E-enriched diet when they were analyzed using an unpaired *t*-test (+*p* = 0.0059). Error bars represent means ± standard error of the mean (*** *p* < 0.0001, CTL SHAM, *n* = 6 vs. CTL INJ, *n* = 6 and VIT E SHAM, *n* = 5 vs. CTL INJ, *n* = 6; *p* > 0.05 CTL SHAM, *n* = 6 vs. VIT E INJ, *n* = 7 and CTL SHAM, *n* = 6 vs. VIT E SHAM, *n* = 5; ** *p* < 0.01, VIT E INJ, *n* = 7 vs. CTL INJ, *n* = 6).

We performed immunohistological analyses to determine the number of oligodendrocytes in the white matter at 7 dpi. Oligodendrocytes were immunodetected using the anti-adenomatosis polyposis coli (APC) antibody. Our results confirmed previous studies showing a significant decrease in the number of oligodendrocytes at one-week post-SCI. Representative images from injured sections labeled with the APC antibody show increased number of APC positive cells in the spinal cord of rats fed the vitamin E diet when compared to controls. Interestingly, the analysis showed that there was an increased number of APC positive cells in the spinal cord of injured rats that were fed the vitamin E diet compared to control injured rats. There was no significant difference between APC positive cells between uninjured rats in the control diet and vitamin E diet. (*p* < 0.05) (Figure 6) (F (3, 26,619) = 18.05, *** *p* < 0.001, CTL SHAM, *n* = 6 vs. CTL INJ, *n* = 6); ** *p* < 0.01, VIT E INJ, *n* = 7 vs. CTL INJ, *n* = 6); *p* > 0.05, CTL SHAM, *n* = 6 vs. VIT E SHAM, *n* = 6). Further statistical analysis showed that there was a significant difference in APC positive cells between uninjured rats in the vitamin E diet and injured vitamin E diet when they were analyzed using an unpaired *t*-test (*p* = 0.0059).

### 3.6. Dietary Vitamin E Upregulates Serotonin Immunoreactivity Following SCI

**Figure 7:**
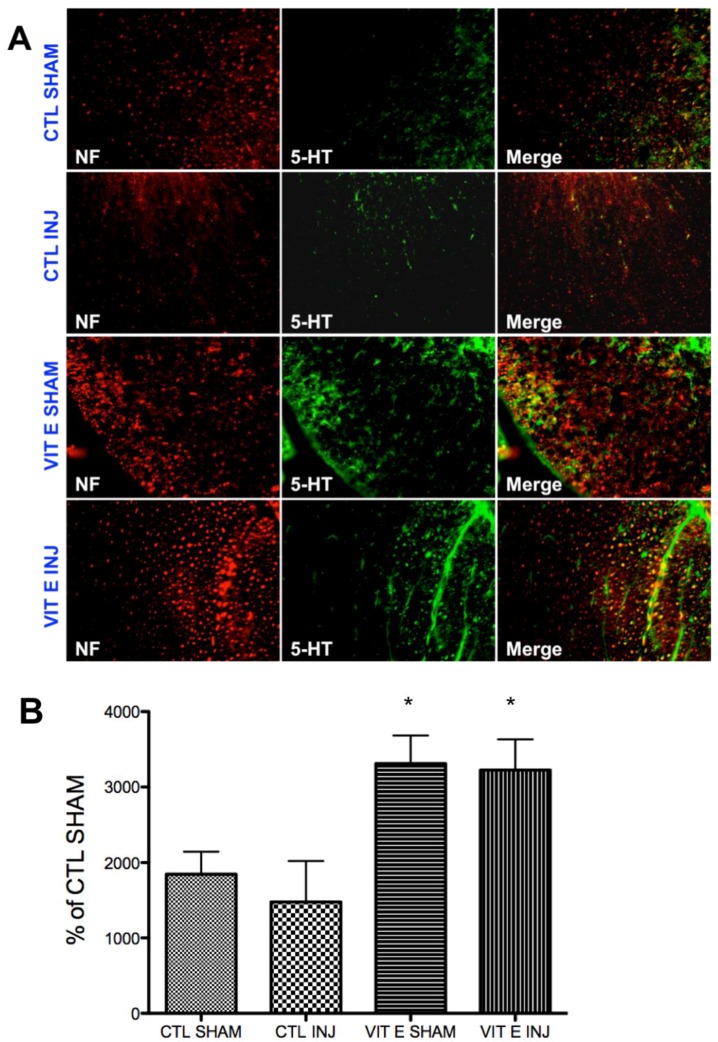
Vitamin E prophylaxis upregulate serotonin immunoreactivity in the white matter at 7 dpi. Serotonin IR was quantified in the white matter at 7 dpi in all four groups (Figure 7A). There was a diet-dependent upregulation of 5-HT-IR in uninjured rats (* *p* < 0.05, CTL SHAM, *n* = 5 vs. VIT E SHAM, *n* = 5) and injured rats (* *p* < 0.05, CTL INJ, *n* = 5 vs. VIT E INJ, *n* = 5) (Figure 7B). There was no difference between uninjured and injured rats in the control diet (*p* > 0.05, CTL SHAM, *n* = 5 vs. CTL INJ, *n* = 5) and between uninjured and injured rats in the vitamin E diet (*p* > 0.05, VIT E SHAM, *n* = 5 vs. VIT E INJ, *n* = 5). 5-HT (5-hydroxytryptamine), NF (neurofilament).

Previous studies have shown decreased serotonin levels after moderate-contusive SCI [64]. Serotonin inhibits afferent transmission and spinal reflexes and plays crucial roles in functional recovery after SCI [65,66,67,68,69,70,71]. Serotonergic signaling is a key mechanism underlying neuronal hyperexcitability after SCI, which has been demonstrated to underlie the pathogenesis of spasticity after SCI [72,73,74,75,76]. Thus, we postulated that vitamin E beneficial effects observed would be consistent with the modulation of the levels of this neurotransmitter. As hypothesized, we found a significant increase in the levels of serotonin in rats fed the vitamin E-enriched diet compared to the control diet in uninjured (*p < 0.05, CTL SHAM, *n* = 5 vs. VIT E SHAM, *n* = 5) and injured rats (*p < 0.05, CTL INJ, *n* = 5 vs. VIT E INJ, *n* = 5) (Figure 7B). Interestingly, we found no significant difference between sham and injured rats (*p* > 0.05, *p* = at least 5 rats).

## 4. Discussion

The present study reports novel findings showing that a two-month chronic dietary supplementation with vitamin E (alpha-tocopherol) improves recovery during the acute phase of SCI and identify potential targets. The significant prophylactic effects of vitamin E supplementation included improved functional locomotor outcomes, accelerated bladder recovery measured in urinary retention time, and reduced hyperreflexia after SCI. Further, the dietary intervention increased numbers of oligodendrocytes and increased supraspinal serotonin IR, indicating potential targets underlying the restorative potential of vitamin E in SCI. 

Spinal cord injury has a devastating effect on affected individuals. Patients experience serious secondary complications such as urinary retention, a sign of autonomic bladder dysfunction, that has serious practical physiological and psychological consequences [77,78]. Thus, the search of alternative complementary interventions with the potential to lessen the effect of this condition is needed. Although there have been several studies considering the effects of vitamin E during spinal cord injury, little progress has been made in developing appropriate evidence-based complementary therapies. Questions needed to be address for further assessment include appropriate dosage, timing of administration and proper use of dietary prophylaxis. Also, while several studies have shown the effects of vitamin E on improvement locomotion and other parameters, these effects on locomotion has not been quantified using the BBB scores and it is unknown whether this complementary treatment is beneficial in addressing urinary retention time, or the H-reflex. To properly evaluate a potential vitamin E effect on these parameters it is important to ensure that the animal is exposed to the proper levels of vitamin E. The current study uses dietary prophylaxis with vitamin E by exposing the animals to a chronic dietary intervention for two months before the animal were subject to the traumatic contusion injury on the cord. A chronic dietary intervention was deemed to be a better approach to avoid potential hurdles associated with vitamin E stability. This approach allowed us to assess with confidence key functional outcomes and determine whether vitamin E had a significant impact. We also proceeded to assess selective functional parameters and quantifying vitamin E effects on locomotion using for the first time the BBB scores. 

The well documented antioxidant actions of vitamin E would be beneficial in physiological process that, while affected by the injury, still have functional connections. In this context, we evaluate whether bladder dysfunction was a good target for this treatment. Current interventions used to reduce bladder dysfunction after SCI include cholinergic muscarinic receptor antagonists [79,80,81,82,83,84,85], chemical blockade of C-fiber afferent neurotransmission with capsaicin or resiniferatoxin [86,87,88,89,90,91,92] and alpha1- acetylcholine receptor (AR) receptor antagonists [93,94,95,96,97,98,99]. Additional interventions include suppression of motorneuron or interneuron excitation in the spinal cord by glycine, GABA agonists, and baclofan is being used to treat dysfunctional contraction of the external urethral sphincter [100,101,102,103,104,105,106]. Botulin toxin, a presynaptic neuromuscular blocker, is now FDA-approved to treat bladder hyperreflexia by inducing reversible muscle weakness [107,108,109,110,111,112]. Various studies support the use of antioxidant therapy to address bladder function following SCI. For instance, treatment with quercetin was shown to improve bladder contractility, while decreasing reactive oxygen species, plasma cytokines, and caspase 3, and prevented depletion of free radical scavengers after SCI in rats [113]. Additional treatment with antioxidant cranberry extract supplements for at least 6 months showed a decrease in urinary tract infections [114]. These approaches to address bladder recovery following SCI suggest that complementary therapies that can improve nerve conduction may be useful as part of comprehensive treatment. In this context, vitamin E could specifically decrease urinary retention through the improvement of nerve conduction. For instance, spinal cord evoked potentials after injury showed greater recovery of both amplitude and latency in a vitamin E supplemented group compared to control [44,48]). Also, in the present study we found that vitamin E supplementation significantly inhibits the H-reflex depression. These findings are consistent with a role of vitamin E in enhancing nerve conductivity in the injured cord which is may also explain the significant increase in the BBB locomotion scores seen in these animals fed with the enriched vitamin E diet reported here. These data expand previous work showing vitamin E improving locomotion when administered in a prophylactic manner [43,44,48] or after injury [45,46]. When compared to vitamin C supplementation after injury, vitamin E was shown to be more effective for locomotion recovery [46]. Proven actions of vitamin E in reducing lipid peroxidation products such as thiobarbituric acid reactive substances [44,48] and malondialdehyde [47] may be responsible at least in part for these effects [36]. Further, vitamin E improves recovery by decreasing ischemia [42] and downregulating arachidonate and prostanoids [41].

The improved outcomes exhibited by the rats that consumed the vitamin E-enriched diet were associated with an increased preservation of oligodendrocytes of the injured cord. Interestingly, this increase of oligodendrocytes survival was not accompany with an increased survival of neurons. This finding is consistent with reports showing the high vulnerability of oligodendrocytes to oxidative stress. For instance, oligodendrocytes and pre-oligodendrocytes have been shown to be highly sensitive to abnormal high levels of intracellular free radicals that follow cysteine deprivation and treatment with vitamin E and other ROS scavengers (ascorbate, idebenone, and *N*-tert-butyl-alpha-phenylnitrone) promote their survival [115,116]. Vitamin E also protects murine oligodendrocytes in culture from ROS generation and apoptosis caused by cytotoxic oxysterols [117,118,119], and from lipid peroxidation in combination with ascorbate acid [120]. Although this was not directly addressed in the present study, a significant increase in oligodendrocytes in the rats that consumed the vitamin E diet may be implicated in the improvement seen on H-reflex depression, bladder reflex recovery, and locomotion in these rats because of myelin and axonal preservation [25,121,122,123,124,125,126]. Therefore, vitamin E may be able to exert its beneficial effects in the event of spinal cord injury even in the absence of neuronal preservation due to increased survival of oligodendrocytes. Future clinical study may be necessary to assess the value of a similar strategy to treat bladder dysfunction and improve nerve conductivity following SCI.

Our results showing a significant upregulation in serotonin levels in rats fed a vitamin E prophylactic diet further strengthens vitamin E as a potential therapeutic agent for SCI complications. This is supported by several studies showing an improved voiding efficiency after SCI following increased catecholaminergic and serotonergic axonal growth [127,128,129,130]. This is consistent with findings showing a direct correlation between low serotonin and abnormal H-reflex depression [64]. Serotonin depletion has also been found to positively correlate with the degree of paralysis and disease severity in rat model for multiple sclerosis [131,132]. In models of SCI regeneration of spinal serotonergic neurons is associated with functional recovery [133,134]. 

In summary, we propose increased survival of oligodendrocytes and upregulation of serotonin levels as potential mechanisms through which dietary vitamin E prophylaxis improves locomotion, H- reflex depression, and bladder recovery in the context of SCI. 

The findings reported herein are consistent with previous studies from our lab showing that administration of antioxidants such as omega-3 fatty acids stimulate recovery following contusion injury to the spinal cord [38,39,40]. To date, several studies have shown the beneficial effects of antioxidants to restore function after trauma. However, studies investigating the effects of dietary vitamin E in neural repair are limited. Our findings suggest the potential of this nutritional-based intervention to ameliorate functional impairments and cell survival following SCI. Furthermore, our study supports the importance of nutrition to ameliorate the augmented state of cellular oxidative stress observed during SCI. 

## 5. Conclusions

In conclusion our study reports provides evidence that a two-month chronic dietary supplementation with vitamin E (alpha-tocopherol) aids in the functional recovery of SCI during the acute phase. Specifically, vitamin E seemed to have a prophylactic effect resulting in improved locomotor outcomes, accelerated bladder recovery measured in urinary retention time, and reduced hyperreflexia. Our data indicates that increased survival of oligodendrocytes and increased supraspinal serotonin IR are potential targets for the underlying mechanisms of vitamin E and its prophylactic effects in the acute phase of SCI. Further studies need to be done to investigate the effects of vitamin E in the chronic phase after SCI.

## Figures and Tables

**Table 1:** Detailed diet composition for Control and vitamin E-Enriched Diet.

Ingredient	AIN-93G Control Diet (%)	AIN-93G Vitamin E
Soybean Oil	7	7
Vitamin E, IU kg	0.0816	51
Total Saturated Fat	1.13 g/100 g	1.00 g/100 g
Total Monosaturated Fat	1.61 g/100 g	1.49 g/100 g
Total Polysaturated Fat	4.09 g/100 g	4.34 g/100 g
Percentage kcal Carbohydrates	64.7	60.5
Percentages kcal Protein	18.8	21.1
Percentage kcal Fat	16.5	18.4
